# Exercise, weight maintenance, and nonalcoholic fatty liver disease risk: a Chinese cohort study

**DOI:** 10.3389/fphys.2024.1359476

**Published:** 2024-03-26

**Authors:** Chao Yang, Peijing Yan, Jiaqi Deng, Yujuan Li, Xia Jiang, Ben Zhang

**Affiliations:** ^1^ Department of Epidemiology and Biostatistics, Institute of Systems Epidemiology, and West China-PUMC C. C. Chen Institute of Health, West China School of Public Health and West China Fourth Hospital, Sichuan University, Chengdu, China; ^2^ School of Public Health, Southwest Medical University, Luzhou, China; ^3^ Department of Ultrasound, The Affiliated Hospital of Southwest Medical University, Luzhou, China; ^4^ Health Management Center, The Affiliated Hospital of Southwest Medical University, Luzhou, China; ^5^ Department of Nutrition and Food Hygiene, West China School of Public Health and West China Fourth Hospital, Sichuan University, Chengdu, China; ^6^ Department of Clinical Neuroscience, Karolinska Institute, Stockholm, Sweden; ^7^ Department of Occupational and Environmental Health, West China School of Public Health and West China Fourth Hospital, Sichuan University, Chengdu, China

**Keywords:** exercise, weight maintenance, nonalcoholic fatty liver disease, mediation analysis, cohort

## Abstract

**Background:** Exercise has been reported to be associated with a reduced risk of nonalcoholic fatty liver disease (NAFLD), but there is no consensus on the role of weight changes in this association. This study aims to investigate whether the impact of exercise on NAFLD is mainly dependent on weight changes or is inherent to exercise itself.

**Methods:** The study recruited 1671 Chinese NAFLD-free adults in 2019, and collected their exercise habits as well as 3 years of medical examination data including anthropometric measurements, blood biochemistry parameters, and liver ultrasound results. Univariate and multivariate logistic regression models were employed to examine the impact of exercise habits on NAFLD risk, with mediation analysis utilized to estimate the magnitude of the role of weight maintenance in the association between exercise and NAFLD.

**Results:** After adjusting for confounders, moderate to high-intensity exercisers were 1.56 times (95% CIs = 1.09–2.22) more likely to successfully control their body weight, and therefore the weight-controlled group had a lower NAFLD risk of 34.9% (95% CIs = 21.8%–56.0%) compared to the weight-gain group. Mediation analysis reveals that exercise can significantly reduce the risk of NAFLD both through weight maintenance (37.1%) and independent of weight maintenance (62.9%).

**Conclusion:** It might be more crucial to emphasize the adoption of regular moderate to high-intensity exercise for preventing NAFLD in the general population, rather than solely focusing on weight maintenance.

## Introduction

Nonalcoholic fatty liver disease (NAFLD) is the most common chronic liver condition characterized by the accumulation of fat in liver cells’ cytoplasm (Bellentani and Marino, 2009), and is considered a manifestation of metabolic syndrome in the liver (Yang and Wang, 2023). In addition to liver-related consequences such as cirrhosis and hepatocellular carcinoma (White et al., 2012), individuals with NAFLD, especially those with nonalcoholic steatohepatitis and fibrosis, are under an elevated risk of cardiovascular disease, cancer, diabetes, and overall death (Kirk et al., 2009; Elias et al., 2010; Haufe et al., 2013; Vilar-Gomez et al., 2015; Ma et al., 2017; Mahfood Haddad et al., 2017; Allen et al., 2018; Allen et al., 2019). Over the past 2 decades, driven by the worldwide obesity epidemic, the global prevalence of NAFLD has significantly increased, rising from 25.3% to 38.0%, leading to a substantial healthcare burden (Younossi et al., 2016; Younossi et al., 2023). Consequently, it is imperative to establish preventive measures to counteract the rapid development of NAFLD.

Previous studies have indicated that exercise is not only associated with a reduced risk of NAFLD but also contributes to its alleviation (Miyake et al., 2015; Orci et al., 2016; Vittorio and Lavine, 2020; Park et al., 2023). However, there is currently no consensus on the mechanisms through which exercise affects NAFLD. A predominant perspective is that the impact of exercise on NAFLD is primarily achieved through weight loss (Zhang et al., 2016; Younossi et al., 2021). A randomized controlled trail (RCT) by Zhang et al. found that both moderate and vigorous exercise were equally effective in reducing intrahepatic triglyceride content; however, this effect appeared to be largely mediated by weight loss (Zhang et al., 2016). Moreover, the America Gastroenterological Association (AGA) also recommends that lifestyle modification through diet and exercise to achieve weight loss is beneficial for all NAFLD patients (Younossi et al., 2021). In contrast, another viewpoint contends that exercise has an independent impact on NAFLD, regardless of weight reduction. A longitudinal study from South Korea demonstrated that moderate to vigorous exercise significantly contributed to a decreased risk of NAFLD, regardless of changes in body weight (Sung et al., 2016). An additional RCT further substantiated these findings, indicating that both moderate and high-intensity exercise effectively reduce NAFLD risk, independently of abdominal adiposity or body mass reduction (Winn et al., 2018). Therefore, we conducted this study to explore whether the impact of exercise on NAFLD is solely mediated by changes in body weight or is also independent of the weight mediation.

## Materials and methods

### Study design and subjects

**FIGURE 1:**
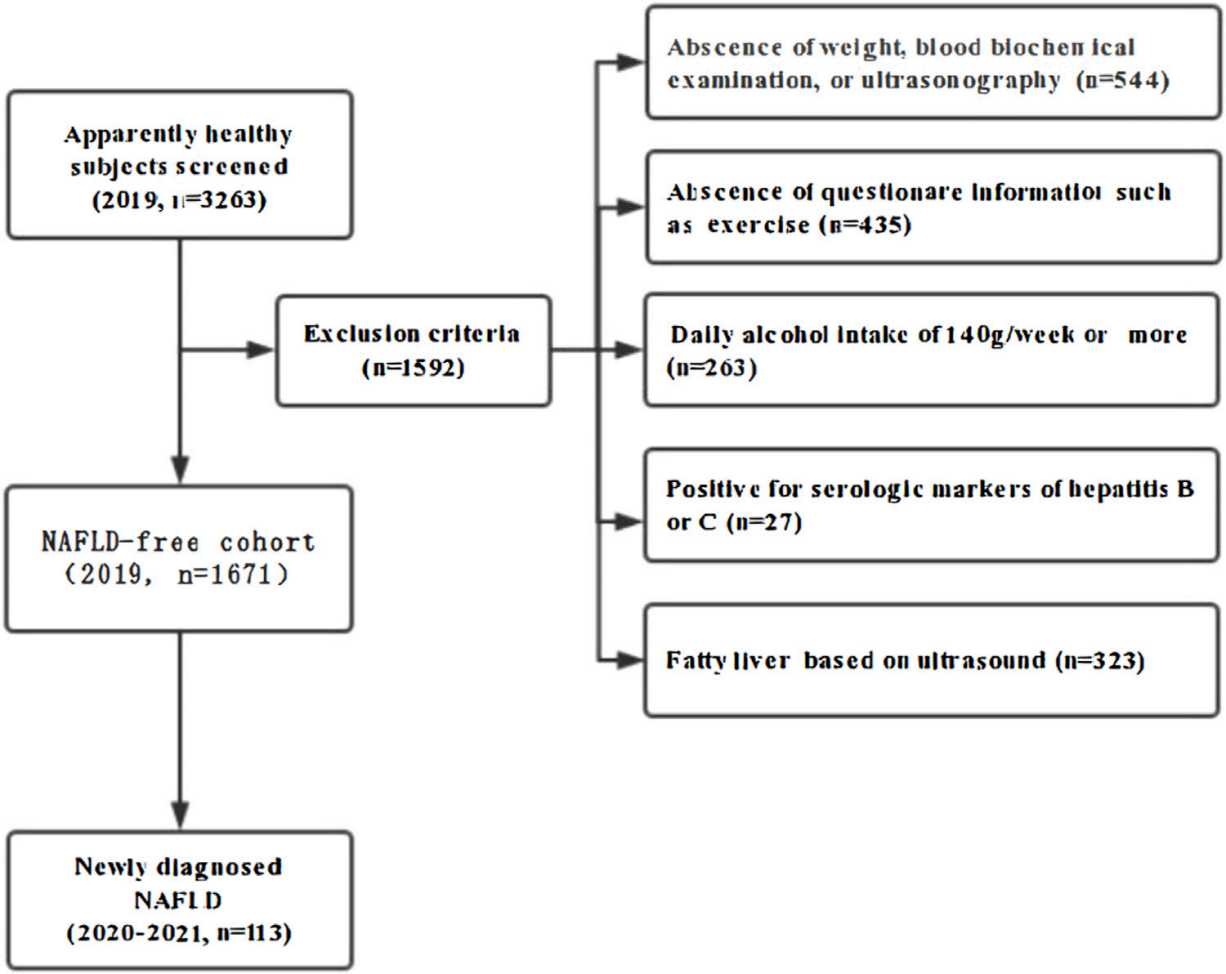
Flow diagram of subjects’ selection. A total of 3,263 adults participated in consecutive annual medical check-ups at the Southwest Medical University Affiliated Hospital. Among these subjects. 1592 individuals were excluded for the following reasons: 544 lacked essential data on weight. Ultrasonography. or blood biochemical examinations: 435 were missing questionnaire responses: 263 were categorized as heavy drinkers. Defined as those consuming 140 g/week or more; 27 tested positive for serologic markers of hepatitis B or C; and 323 were diagnosed with fatty liver based on ultrasonography in 2019. Ultimately. 1671 initially NAFLD-free subjects comprised this cohort study and were retrospectively observed for the development of NAVLD for 2 years.

In 2019, a total of 3263 healthy hospital staff who underwent medical examinations at the affiliated hospital of Southwest Medical University were enrolled in the study, all of whom participated in annual check-ups for three consecutive years (2019-2021). Among these subjects, 1592 individuals were excluded for the following reasons: 544 lacked essential data on weight, ultrasonography, or blood biochemical examinations; 435 were missing questionnaire responses; 263 were categorized as heavy drinkers, defined as those consuming 140 g/week or more; 27 tested positive for serologic markers of hepatitis B or C; and 323 were diagnosed with fatty liver based on ultrasonography in 2019. Ultimately, 1671 initially NAFLD-free subjects comprised this cohort study and were retrospectively observed for the development of NAFLD for 2 years (Figure 1).

### Baseline examinations

Baseline examinations included a health habit inventory, anthropometric measurements, hepatic ultrasonic examination and biochemical measurements.

Systolic blood pressure (SBP), diastolic blood pressure (DBP), and pulse rate were assessed utilizing an automated sphygmomanometer with the individual seated. Height while standing and body weight were gauged with the subject not wearing footwear or outer clothing. Body mass index (BMI, kg/m^2^) was computed as the weight in kilograms divided by the square of the height in meters. The examinations were administered between 8:00–11:00 a.m. Participants were instructed to fast for at least 12 h prior to the examination and to refrain from exercise the day before. The biochemical parameters measured included levels of albumin (ALB), globulin (GLB), direct bilirubin (DBIL), indirect bilirubin (IBIL), triglycerides (TG), high-density lipoprotein cholesterol (HDL-c), low-density lipoprotein cholesterol (LDL-c), fasting blood glucose (FBG), creatinine (Cr), blood urea nitrogen (BUN), serum uric acid (SUA), aspartate aminotransferase (AST), alanine aminotransferase (ALT), and homocysteine (HCY). All biochemical parameters were measured by an automatic biochemical analyzer (BS-2200M).

### Assessment of outcomes and definitions

Hypertension was defined as either self-reported use of antihypertensive medication or having a systolic blood pressure greater than or equal to 140 mmHg, or a diastolic blood pressure of ≥90 mmHg.

Participants’ exercise status was ascertained through the query: “Have you exercised more than 30 min per week in the past 3 months?” For those who select “yes, ”further questions concerning the sort of exercise were asked in order to gauge its intensity. Low-intensity exercise was characterized by maintaining a heart rate between 100–120 beats per minute, with a slight increase in body warmth, typically exemplified by activities like slow walking. Medium-intensity exercise, on the other hand, involved sustaining a heart rate of 130–150 beats per minute, noticeable sweating, faster breathing, and a moderate sense of exertion, as observed in activities such as brisk walking, jogging, bicycling, or tai chi. High-intensity exercise was described by a heart rate ranging from 160–170 beats per minute, accompanied by sweating and heavy breathing, and was often associated with activities like basketball, soccer or swimming.

Weight change values were computed by subtracting the weight in 2019 from the weight in 2021, and weight maintenance was defined as a weight gain not exceeding 2% of the 2019 weight, which was further adjusted to a weight gain of no more than 1% when conducting sensitivity analysis in the Supplementary Material.

The diagnosis of fatty liver was based on the results of abdominal ultrasonography conducted using a GE LOGIQ E9 sonography machine equipped with a 2.0–5.0 MHz probe. Ultrasound examinations were performed by senior imaging specialists. Hepatic steatosis was diagnosed based on characteristic echo patterns in accordance with conventional criteria. These patterns included evidence of diffuse hyperechogenicity of the liver relative to the kidneys, ultrasound beam attenuation, and poor visualization of intrahepatic structures (Targher et al., 2005). NAFLD was diagnosed by abdominal ultrasonography, and exclusion criteria included excessive alcohol use (greater than 140 g per week) or viral infections (Jian-gao and Chinese Liver Disease, 2010).

### Statistical analyses

Continuous variables were expressed as means ± standard deviations or medians with upper and lower quartiles, and were compared using one-way analysis of variance (ANOVA) or the Kruskal-Wallis test, depending on the normality of data. Categorical variables were described in terms of the number of cases and composition ratios, and were compared using the chi-square test. Univariate and multivariate logistic regression models were used to analyze the effect of exercise habits on NAFLD risk. Model fitting was performed using the enter method, and confounding variables including age, gender, and blood biochemical parameters were incorporated into the multivariate model. Furthermore, to ascertain whether weight maintenance serves as a mediator, additional analyses were conducted to assess the effect of exercise habits on weight maintenance as well as the impact of weight maintenance on the risk of NAFLD. The indirect effects of the exercise habit on NAFLD risk (via weight maintenance) were calculated using the product method, but results of the difference method are also provided (Cashin et al., 2023). As a sensitivity study, all comparable analyses were re-performed after lowering the weight maintenance criteria from no more than 2%–1%. All statistical analyses were performed using the SPSS software package version 25.0 for Windows. *p* < 0.05 (2-tailed) was considered as statistically significant.

## Results

### Baseline characteristics

At baseline, the mean age of the 1671 participants was 33.2 years, of which 338 (20.2%) were males. Subjects were categorized into three groups based on their exercise habits: the non-exercise group, the low-intensity exercise group, and the moderate to high-intensity exercise group. Except for the prevalence of hypertension, levels of ALB, DBIL, TG, and FBG, statistically significant differences were observed among the three groups in all other baseline characteristics. Generally, individuals habitually engaging in moderate to high-intensity exercise were older, had higher BMI and WC, lower pulse rate, and elevated biochemical markers compared to those in the non-exercise or low-intensity exercise groups (Table 1).

**TABLE 1:** Baseline characteristics of populations with different exercise habits.

Variable	All *n* = 1671	Non-exercise *n* = 260	Low-intensity *n* = 1006	Moderate to high-intensity *n* = 405	*P*
Age (year)	33.2 ± 8.4	30.3 ± 5.8	33.3 ± 8.6	34.8 ± 8.8	<0.001^a^
Gender, male/female	338/1333	32/228	169/837	137/268	<0.001^b^
BMI (kg/m^2^)	21.7 ± 2.5	21.1 ± 2.5	21.5 ± 2.4	22.4 ± 2.6	<0.001^a^
WC (cm)	73.9 ± 7.5	72.3 ± 7.2	73.7 ± 7.1	75.4 ± 8.2	<0.001^a^
Hypertension, *n* (%)	84 (5.0)	11 (4.2)	46 (4.6)	27 (6.7)	0.220^b^
Pulse rate	82.6 ± 11.8	83.6 ± 12.2	83.2 ± 12.0	80.3 ± 10.9	<0.001^a^
ALT (μmol/L)	14.0 (10.7, 19.6)	12.8 (9.9, 18.3)	13.9 (10.6, 19.2)	15.1 (11.6, 21.2)	<0.001^c^
AST (μmol/L)	18.1 (15.5, 21.5)	17.5 (15.1, 20.4)	17.9 (15.4, 21.4)	19.2 (16.1, 22.8)	<0.001^c^
ALB (g/dL)	46.2 ± 2.5	46.3 ± 2.8	46.1 ± 2.4	46.2 ± 2.5	0.359^a^
GLB (g/dL)	30.1 ± 3.0	29.8 ± 2.8	30.3 ± 3.1	29.8 ± 2.9	0.005^a^
DBIL (μmol/L)	4.6 (3.6, 5.9)	4.6 (3.4, 5.7)	4.6 (3.6, 5.8)	4.7 (3.8, 6.1)	0.164^c^
IBIL (μmol/L)	7.5 (6.0, 9.5)	7.3 (5.6, 9.0)	7.5 (6.1, 9.4)	7.9 (6.0, 10.3)	0.006^c^
BUN (mmol/L)	4.6 ± 1.2	4.4 ± 1.1	4.6 ± 1.1	4.9 ± 1.4	<0.001^a^
SUA (mmol/L)	283.5 ± 67.4	270.6 ± 57.6	279.5 ± 66.0	302.1 ± 73.2	<0.001^a^
Cr (μmol/L)	55.9 ± 11.2	52.8 ± 9.7	55.1 ± 10.6	60.0 ± 12.6	<0.001^a^
TG (mmol/L)	0.82 (0.62, 1.13)	0.79 (0.63, 1.03)	0.81 (0.61, 1.15)	0.85 (0.63 1.16)	0.383^c^
HDL-c (mmol/L)	1.57 ± 0.36	1.62 ± 0.32	1.58 ± 0.37	1.53 ± 0.37	0.034^a^
LDL-c (mmol/L)	2.56 ± 0.79	2.46 ± 0.64	2.56 ± 0.76	2.64 ± 0.93	0.016^a^
FBG (mmol/L)	4.84 ± 0.42	4.80 ± 0.39	4.84 ± 0.42	4.86 ± 0.44	0.224^a^
HCY (μmol/L)	8.1 (6.7, 9.5)	7.8 (6.3, 9.3)	8.1 (6.7, 9.4)	8.4 (7.0, 10.0)	0.003^c^

^a^ One way ANOVA, is used for analyzing quantitative data following a normal distribution.

^b^ Chi-square test was used for analyzing categorical data.

^c^ Kruskal-Wallis test was used for analyzing ranked data.

BMI, body mass index; WC, waist circumference; ALT, alanine transaminase; AST, aspartate transaminase; ALB, albumin; GLB, globulin; DBIL, direct bilirubin; IBIL, indirect bilirubin; BUN, blood urea nitrogen; SUA, serum uric acid; Cr, creatinine; TG, triglyceride; HDL-c, high density lipoprotein cholesterol; LDL-c, low density lipoprotein cholesterol; FBG, fasting blood glucose; HCY, homocysteine.

### Exercise and weight maintenance

Individuals with a regular habit of moderate to high-intensity exercise experienced an average weight reduction of 0.16% over a 2-year period, whereas those who never engaged in exercise and those who participated in low-intensity exercise observed weight gains of 1.78% and 1.58%, respectively, with statistically significant differences. The proportions of individuals who experienced a weight increase of less than 2% over the 2-year period were 52.3% for non-exercisers, 56.8% for low-intensity exercisers, and 66.6% for moderate to high-intensity exercisers. A clear increasing trend in weight maintenance rates with exercise intensity was observed (*P* for trend <0.001). The likelihood of successful weight maintenance for moderate to high-intensity exercisers was 1.82 times higher compared to non-exercisers in crude model (model 1). After adjusting for confounders, this value decreased to 1.56 but remained statistically significant. Furthermore, an overall increasing trend of control rates with higher exercise intensity was also observed (Table 2). The impact of moderate to high-intensity exercise on weight maintenance remained consistent after changing the threshold from 2% to not exceeding 1% of the baseline body weight (Supplementary Table S3).

**TABLE 2:** Effect of exercise on weight change and weight maintenance.

Variable	Non-exercise *n* = 261	Low-intensity *n* = 1005	Moderate to high-intensity *n* = 405	*P*	*P* for trend
Weight gain (kg)	0.75 ± 4.72	0.75 ± 4.69	−0.24 ± 4.47	0.001	-
Weight gain, %	1.78 ± 8.19	1.58 ± 8.38	−0.16 ± 7.08	0.001	-
Weight maintenance n, %	136 (52.3)	571 (56.8)	269 (66.6)	<0.001	<0.001
Model 1	1.00 (reference)	1.20 (0.91, 1.57)	1.82 (1.32, 2.50)	<0.001	<0.001
Model 2	1.00 (reference)	1.09 (0.82, 1.43)	1.58 (1.14, 2.20)	0.005	0.003
Model 3	1.00 (reference)	1.06 (0.79, 1.40)	1.51 (1.08, 2.12)	0.013	0.009
Model 4	1.00 (reference)	1.05 (0.78, 1.41)	1.56 (1.09, 2.22)	0.010	0.009

Binary logistic regression was employed for model fitting, with between-group effect size represented by the odds ratio (OR) along with its 95% confidence interval (CI).

Model 1: did not adjust for confounding factors.

Model 2: adjusted for age and gender.

Model 3: model 2 adjustments plus adjustment for body mass index and waist circumference.

Model 4: model 3 adjustments plus adjustment for hypertension, pulse rate, alanine transaminas, aspartate transaminase, albumin, globulin, direct bilirubin, indirect bilirubin, blood urea nitrogen, serum uric acid, creatinine, triglycerides, high-density lipoprotein cholesterol, low-density lipoprotein cholesterol, fasting blood glucose, and homocysteine.

### Weight maintenance decreases the NAFLD risk

Between 2019 and 2021, individuals who experienced a weight gain of no more than 2% (weight maintenance group) had a NAFLD incidence rate of 5.6%, significantly lower than the incidence of 8.4% observed in those who gained more than 2% of their weight (weight gain group) (*p* = 0.029). The risk of NAFLD in weight maintenance group was only 65.5% (95% CI: 44.7%–96.0%) of that in the weight gain group. After controlling for confounding factors, the weight maintenance group exhibited a NAFLD risk of only 34.9% (95% CI: 21.8%–56.0%) compared to the weight gain group (Table 3). After altering the weight maintenance criteria to not exceeding 1% of the baseline weight, its effectiveness in reducing the risk of NAFLD remained robust (Supplementary Table S4).

**TABLE 3:** Weight maintenance and the risk of NAFLD.

Variable	Weight gain group *n* = 694	Weight maintenance group *n* = 976	*P*
NAFLD *n*, %	58 (8.4)	55 (5.6)	0.029
Model 1	1.00 (reference)	0.66 (0.45, 0.96)	0.030
Model 2	1.00 (reference)	0.58 (0.39, 0.86)	0.006
Model 3	1.00 (reference)	0.35 (0.22, 0.54)	<0.001
Model 4	1.00 (reference)	0.35 (0.22, 0.56)	<0.001

Binary logistic regression was employed for model fitting, with between-group effect size represented by the odds ratio (OR) along with its 95% confidence interval (CI).

Model 1: did not adjust for confounding factors.

Model 2: adjusted for age and gender.

Model 3: model 2 adjustments plus adjustment for body mass index and waist circumference.

Model 4: model 3 adjustments plus adjustment for hypertension, pulse rate, alanine transaminas, aspartate transaminase, albumin, globulin, direct bilirubin, indirect bilirubin, blood urea nitrogen, serum uric acid, creatinine, triglycerides, high-density lipoprotein cholesterol, low-density lipoprotein cholesterol, fasting blood glucose, and homocysteine.

### Exercise decreases the risk of NAFLD

The incidence rates of NAFLD within a 2-year period for individuals who never exercised, engaged in low-intensity exercise, and participated in moderate to high-intensity exercise were 6.9%, 7.7% and 4.4%, respectively. Univariate analysis revealed no statistically significant differences among the three groups. However, in several multivariable models (models 2–4) adjusted for different numbers of confounding factors, both non-exercise group and low-intensity group exhibited significantly higher risks of developing NAFLD compared to the moderate to high-intensity group. In Model 4, these risks were 2.42-fold and 2.79-fold higher, respectively. Furthermore, after additional adjustment for weight maintenance, the low-intensity group still had a higher NAFLD risk compared to the moderate to high-intensity group (2.43-fold), while no statistically significant difference was observed between non-exercise group and moderate to high-intensity group. Across all multivariable models, it was consistently observed that as exercise intensity increased, there was a decreasing trend in NAFLD risk (Table 4). After modifying the weight maintenance criteria to no more than 1%, the analysis results remained consistent (Supplementary Table S5).

**TABLE 4:** The effect of exercise on the risk of NAFLD.

Variable	Moderate to high-intensity *n* = 405	Non-exercise *n* = 261	Low-intensity *n* = 1005	*P*	*P* for trend
NAFLD *n*, %	18 (4.4)	18 (6.9)	77 (7.7)	0.094	0.126
Model 1	1.00 (reference)	1.60 (0.82, 3.13)	1.78 (1.05, 3.02)	0.099	0.127
Model 2	1.00 (reference)	2.12 (1.06, 4.23)	2.04 (1.20, 3.49)	0.027	0.019
Model 3	1.00 (reference)	2.63 (1.19, 5.82)	3.10 (1.71, 5.63)	0.001	0.004
Model 4	1.00 (reference)	2.42 (1.05, 5.56)	2.79 (1.49, 5.23)	0.006	0.014
Model 5	1.00 (reference)	2.16 (0.93, 5.02)	2.43 (1.29, 4.59)	0.023	0.037

Binary logistic regression was employed for model fitting, with between-group effect size represented by the odds ratio (OR) along with its 95% confidence interval (CI).

Model 1: did not adjust for confounding factors.

Model 2: adjusted for age and gender.

Model 3: model 2 adjustments plus adjustment for body mass index and waist circumference.

Model 4: model 3 adjustments plus adjustment for hypertension, pulse rate, alanine transaminas, aspartate transaminase, albumin, globulin, direct bilirubin, indirect bilirubin, blood urea nitrogen, serum uric acid, creatinine, triglycerides, high-density lipoprotein cholesterol, low-density lipoprotein cholesterol, fasting blood glucose, and homocysteine.

Model 5: model 4 adjustments plus adjustment for weight maintenance.

### Association between exercise, weight maintenance and NAFLD risk

**FIGURE 2:**
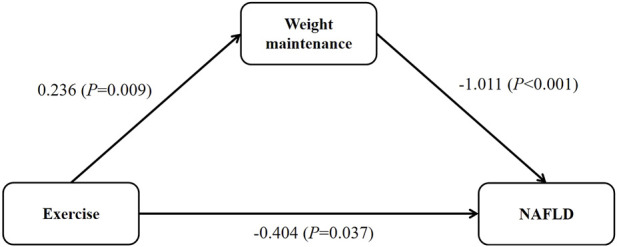
The relationship between exercise, weight maintenance and NAFLD.

Summarizing the relationship among exercise habits, weight maintenance, and NAFLD risk, as depicted in Figure 2, with weight maintenance serving as a mediator. Exercise contributed to weight maintenance (0.236, *p* = 0.009), weight maintenance led to a reduction in NAFLD risk (−1.011, *p* < 0.001), and exercise had a direct impact on lowering NAFLD risk (−0.404, *p* = 0.037). Consequently, employing the product method, the indirect effect of exercise habits on NAFLD risk through weight maintenance was calculated as −0.239 (0.236 −1.011), accounting for 37.1% of the total effect. Thus, exercise habit had a 62.9% direct impact on NAFLD risk. In contrast, utilizing the difference method, the indirect effect was found to constitute 12.9% of the total effect (Supplementary Table S2). When exercise habits were incorporated into the model as dummy variables, with moderate to high-intensity exercise serving as the reference, similar results were obtained (Supplementary Tables S1, S2; Supplementary Figure S1). In the sensitivity analysis with adjusted weight maintenance criteria, consistent results were obtained. The direct effect of exercise and the indirect effect through weight maintenance were 67.7% and 32.3%, respectively. (Supplementary Tables S6, S7; Supplementary Figure S2).

## Discussion

The relationship between exercise, weight change, and NAFLD has been extensively investigated, yet results remain inconclusive. This study reveals that individuals engage in moderate to high-intensity exercise, despite being older or with higher BMI, waist circumference, and various biochemical markers, exhibit a significantly lower risk of developing NAFLD within a 2-year period. Exercise can effectively reduce the risk of NAFLD both indirectly, through its impact on weight maintenance, and directly, independent of weight maintenance. Notably, the indirect effect accounts for only approximately 37% of the overall impact of exercise, which is less significant than the direct effect of exercise. Through mediation analysis, our study offers a fresh perspective for a better understanding of the role of exercise in improving NAFLD.

This study identified an intriguing phenomenon: individuals with a habit of moderate to high-intensity exercise at baseline exhibited poorer metabolic profiles, such as higher BMI, TG levels, and lower HDL levels, among others. This could be attributed to the fact that individuals in the moderate to high-intensity exercise group tended to be older. On the other hand, their inclination towards moderate to high-intensity exercise might also be influenced by their less healthy metabolic profiles. However, despite having worse metabolic conditions, individuals engaging in moderate to high-intensity exercise demonstrated a lower risk of developing NAFLD. Through multivariable logistic regression analysis, we adjusted for confounding factors at baseline, including age, BMI, and various metabolic indicators, and found that moderate to high-intensity exercise was associated with a reduced risk of NAFLD. This underscores the potential preventive role of regular exercise habits in NAFLD.

As weight loss is often linked to a decrease in liver fat, offering the potential to reverse disease progression (Elias et al., 2010; Vilar-Gomez et al., 2015), a plethora of studies have emphasized the role of weight reduction in improving NAFLD (Hannah and Harrison, 2016; Katsagoni et al., 2018; Younossi et al., 2021). In the meanwhile, exercise, like food restriction, is frequently recognized as an approach of weight reduction or weight management (Younossi et al., 2021). However, shedding excess weight can be a formidable task, particularly for seemingly healthy individuals. Our present study indicated that apart from its impact on weight, exercise by itself considerably decreases the risk of NAFLD. This is supported by the results of a cohort study with 233,676 participants which found that regardless of alterations in body weight, weekly exercise with a frequency of 1–2 or more times was associated with a decreased risk of NAFLD in the general population (Sung et al., 2016). In addition, another study on individuals after NAFLD remission suggested that, in addition to weight gain, lack of exercise was also a risk factor for NAFLD relapse (Nakanishi et al., 2021). This corroborates the notion that exercise can affect essential metabolic and inflammatory mechanisms that are linked to the development and progression of NAFLD (Richter and Ruderman, 2009). By increasing peroxisome proliferator-activated receptors and adiponectin levels, exercise has the potential to ameliorate hepatic steatosis, leading to improved insulin resistance and enhanced lipolysis (Petridou et al., 2007; Guo et al., 2015). Insights from another study may help us better understand the mechanisms by which exercise affects NAFLD (Colosimo et al., 2023). It highlighted that both metabolic flexibility and insulin resistance play crucial roles in the development of NAFLD through distinct mechanisms. Regular physical activity promotes metabolic flexibility by increasing energy expenditure and facilitating efficient switching between different energy substrates, thereby aiding in the prevention and alleviation of NAFLD. In contrast, weight loss is effective in improving insulin sensitivity but has no effect on metabolic flexibility. To conclude, it can be affirmed that exercise exerts an independent effect on NAFLD risk.

Despite numerous studies examining the impact of exercise and weight changes on NAFLD, few have analyzed the direct effect of exercise on NAFLD as well as its indirect effects through weight changes. In this study, we observed that exercise demonstrates both direct and indirect effects on the development of NAFLD in the general population, with the direct impact being predominant (62.9% vs. 37.1%). These findings underscore the increasing significance of exercise in the general population, advocating exercise without an excessive focus on weight maintenance may also effectively prevent the onset of NAFLD.

Furthermore, our study suggests that only moderate to high-intensity exercise effectively reduces NAFLD risk, whether mediated through weight maintenance or acting independently. Preceding this, several studies have explored the connection between the intensity of exercise and NAFLD. For instance, Kistler et al. reported in a cross-sectional study that NAFLD patients engaging in vigorous physical activity had a lower prevalence of biopsy-confirmed hepatic fibrosis compared to those involved in moderate physical activity, highlighting the role of exercise intensity (Kistler et al., 2011). Similarly, a meta-analysis (Katsagoni et al., 2017) concluded that high-intensity interval exercise training may be more effective in reducing liver steatosis than exercise training of low-to-moderate intensity. In line with these findings, AGA recommends NAFLD patients to engage in 150–300 min of moderate-intensity or 75–150 min of high-intensity exercise per week (Hashida et al., 2017; Younossi et al., 2021). Up to this point, RCT studies investigating the relationship between exercise and NAFLD have predominantly targeted patients and obese individuals. Further randomized controlled research is warranted to establish exercise recommendations for the general population.

This study has several limitations that deserve attention. Firstly, the diagnosis of NAFLD relied on liver ultrasound tests, which, although commonly used, are operator-dependent and have lower sensitivity. In our study, among 1671 individuals without NAFLD at baseline, 113 developed NAFLD within 2 years, indicating an annual incidence rate of approximately 3.4%. This rate is lower than that reported in another cohort study using magnetic resonance imaging diagnosis (median follow-up time of 4.6 years, cumulative incidence rate of 26.5%, and annual incidence rate of approximately 5.8%) (Chen et al., 2023). This discrepancy may be attributed to the lower sensitivity of ultrasound. However, it's noteworthy that our study participants were significantly younger (33.2 vs. 61.6 years), which could potentially confound this observation. Secondly, it was conducted as a retrospective cohort study, utilizing medical examination data and relying on exercise information obtained through self-reported surveys. This approach, while practical for large-scale studies, may have compromised its evidential robustness when compared to RCTs. Thirdly, participants’ exercise habits may change over time, potentially confounding the study results. However, evidence shows that exercise behavior is moderately to highly stable throughout the lifespan, particularly among adults (van der Zee et al., 2019). Furthermore, our follow-up period lasted only 2 years. As a result, it might be argued that this impact is to some extent acceptable. Fourthly, the relatively modest sample size in this study might have led to insufficient statistical power, increasing the chances of non-significant findings in between-group comparisons. Notably, while the study indicated that individuals engaged in moderate to high-intensity exercise had a reduced risk of NAFLD in comparison to those involved in low-intensity exercise, it is important to acknowledge that this difference did not attain statistical significance when compared to the non-exercise group. This could be attributed to the limited sample size. Finally, due to practical constraints, dietary information was not collected in the study, precluding an assessment of its impact.

## Conclusion

In the general population, regular moderate to high-intensity exercise independently reduces the risk of NAFLD, with its benefits outweigh those derived from weight maintenance. Therefore, rather than focusing specifically on weight maintenance, promoting routine moderate to high-intensity exercise may be a more beneficial approach for preventing NAFLD.

## Data Availability

The raw data supporting the conclusion of this article will be made available by the authors, without undue reservation.

## Abbreviations

NAFLD, nonalcoholic fatty liver disease; RCT, Randomized Controlled Trail; AGA, America Gastroenterological Association; SBP, systolic blood pressure; DBP, diastolic blood pressure; BMI, body mass index; WC, waist circumference; ALT, alanine transaminase; AST, aspartate transaminase; ALB, albumin; GLB, globulin; DBIL, direct bilirubin; IBIL, indirect bilirubin; BUN, blood urea nitrogen; SUA, serum uric acid; Cr, creatinine; TG, triglyceride; HDL-c, high density lipoprotein cholesterol; LDL-c, low density lipoprotein cholesterol; FBG, fasting blood glucose; HCY, homocysteine; ANOVA, one-way analysis of variance; OR, odds ratio; CI, confidence interval.
