# Supplementary material for: Exercise, weight maintenance, and nonalcoholic fatty liver disease risk: a Chinese cohort study
Source: Front Physiol. 2024 Mar 26;15:1359476. doi: 10.3389/fphys.2024.1359476 (PMC11002183; doi:10.3389/fphys.2024.1359476)
Supplement: Supplementary file 1 [file DataSheet1.docx]

**Supplementary material**

**The detailed results of the calculations for the mediation effects using both methods are presented in Table S1 and Table S2.**

**Table S1 effect parameters calculated by product method**

| **Comparison** | **β_(X-M)_** | **β_(M-Y)_** | **β_(X-Y)_**  **Indirect effect** | **Direct effect** | **β_(X-Y)_**  **Total effect** | **Direct effect %** | **Indirect effect %** |
| --- | --- | --- | --- | --- | --- | --- | --- |
| non-exercise vs. moderate to high | -0.443 | -0.970 | 0.430 | 0.772 | 1.202 | 64.2 | 35.8 |
| low vs. moderate to high | -0.393 | -0.970 | 0.381 | 0.888 | 1.269 | 70.0 | 30.0 |
| no dummy variables | 0.236 | -1.011 | -0.239 | -0.404 | -0.643 | 62.9 | 37.1 |

**Table S2 effect parameters calculated by difference method**

| **Comparison** | **β_(X-M)_** | **β_(M-Y)_** | **β_(X-y)_**  **Indirect effect** | **Direct effect** | **β_(X-y)_**  **Total effect** | **Direct effect %** | **Indirect effect %** |
| --- | --- | --- | --- | --- | --- | --- | --- |
| non-exercise vs. moderate to high | -0.443 | -0.970 | 0.111 | 0.772 | 0.883 | 87.4 | 12.6 |
| low vs. moderate to high | -0.393 | -0.970 | 0.138 | 0.888 | 1.026 | 86.5 | 13.5 |
| no dummy variables | 0.236 | -1.011 | -0.060 | -0.404 | -0.464 | 87.1 | 12.9 |


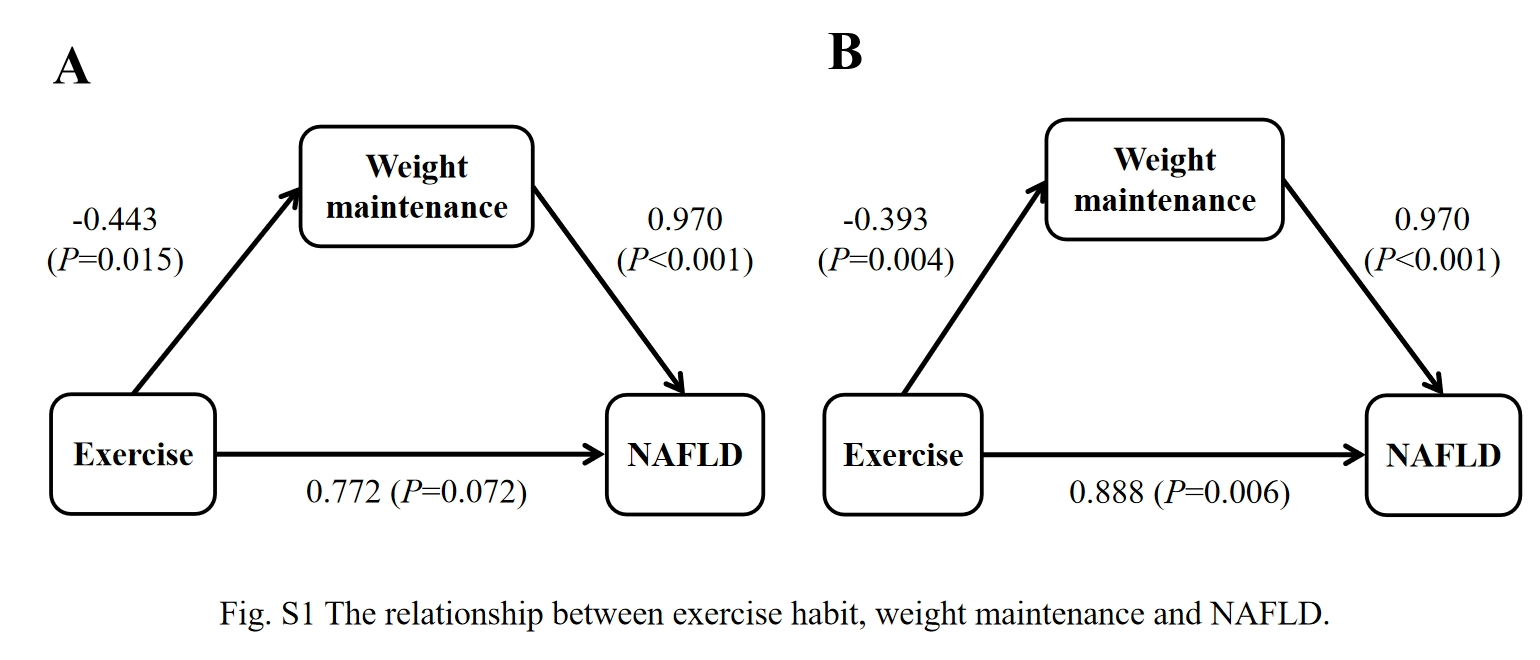


A. The mediation effect diagram comparing non-exercisers to individuals engaged in moderate to high-intensity exercise. B. The mediation effect diagram comparing low-intensity exercise to individuals engaged in moderate to high-intensity exercise.

**We modified the criterion for weight maintenance from an increase of no more than 2% to 1% as a sensitivity analysis, and the results are as follows. We have highlighted discrepancies from the main analysis results in blue font.**

**Table S3** provides information on the impact of exercise habits on weight maintenance, indicating that moderate to high-intensity exercise continues to contribute to weight maintenance. Table S4 presents the effects of weight maintenance on the risk of NAFLD, with the analysis results consistently indicating its role in reducing the risk. Table S5 illustrates the impact of exercise habits on the risk of NAFLD, showing that individuals engaged in low-intensity exercise still have a higher risk of NAFLD compared to those participating in moderate to high-intensity exercise. This highlights the beneficial effect of exercise in reducing NAFLD risk. Figure S2 illustrates that weight maintenance remains a mediating factor between exercise habits and the risk of NAFLD, contributing partially to mediation. The indirect effects calculated using the product method and the difference method are 32.3% and 12.5%, respectively. Table S6 and S7 provide a detailed presentation of the calculation process for both methods.

**Table S3 Effect of exercise on weight change and weight maintenance**

| **Variable** | **No exercise *n*=260** | **Low**  ***n*=1006** | **Moderate to high *n*=405** | ***P*** | ***P* for trend** |
| --- | --- | --- | --- | --- | --- |
| Weight gain (kg) | 0.75±4.72 | 0.75±4.69 | -0.24±4.47 | 0.001 | － |
| Weight gain, % | 1.78±8.19 | 1.58±8.38 | -0.16±7.08 | 0.001 | － |
| weight maintenance n, % | 115(44.2) | 504(50.1) | 226(58.3) | 0.001 | <0.001 |
| Model 1 | 1 | 1.266(0.962, 1.665) | 1.761(1.286, 2.411) | 0.001 | <0.001 |
| Model 2 | 1 | 1.175(0.890, 1.550) | 1.602(1.160, 2.214) | 0.008 | 0.003 |
| Model 3 | 1 | 1.132(0.851, 1.508) | 1.522( 1.090, 2.126) | 0.023 | 0.009 |
| Model 4 | 1 | 1.161(0.862, 1.563) | 1.539(1.083, 2.186) | 0.035 | 0.012 |

Model 1 did not adjust for confounding factors. Model 2 adjusted for age and gender. Model 3 adjusted for body mass index and waist circumference on the basis of Model 2. Model 4 adjusted for hypertension, pulse rate, alanine transaminas, aspartate transaminase, albumin, globulin, direct bilirubin, indirect bilirubin, blood urea nitrogen, serum uric acid, creatinine, triglycerides, high-density lipoprotein cholesterol, low-density lipoprotein cholesterol, fasting blood glucose, and homocysteine, on the basis of Model 3.

**Table S4 weight maintenance and the risk of NAFLD**

| **Variable** | **Weight gain group**  ***n*=816** | **weight maintenance group**  ***n*=855** | ***P*** |
| --- | --- | --- | --- |
| NAFLD *n*, % | 64(7.8) | 49(5.7) | 0.086 |
| Model 1 | 1 | 0.714(0.486, 1.050) | 0.087 |
| Model 2 | 1 | 0.653(0.442, 0.967) | 0.033 |
| Model 3 | 1 | 0.409(0.262, 0.640) | <0.001 |
| Model 4 | 1 | 0.401(0.251, 0.641) | <0.001 |
| Model 5 | 1 | 0.429(0.268, 0.688) | <0.001 |

Model 1 did not adjust for confounding factors. Model 2 adjusted for age and gender. Model 3 adjusted for body mass index and waist circumference on the basis of Model 2. Model 4 adjusted for hypertension, pulse rate, alanine transaminas, aspartate transaminase, albumin, globulin, direct bilirubin, indirect bilirubin, blood urea nitrogen, serum uric acid, creatinine, triglycerides, high-density lipoprotein cholesterol, low-density lipoprotein cholesterol, fasting blood glucose, and homocysteine, on the basis of Model 3; Model 5 adjusted for physical exercise habit in addition to the variables adjusted for in Model 4.

**Table S5 The effect of exercise habit on the risk of NAFLD**

| **Variable** | **Moderate to high-intensity**  ***n*=405** | **No exercise**  ***n*=260** | **Low-intensity**  ***n*=1006** | ***P*** | ***P* for trend** |
| --- | --- | --- | --- | --- | --- |
| NAFLD *n*, % | 18(4.4) | 18(6.9) | 77(7.7) | 0.094 | 0.126 |
| Model 1 | 1 | 1.599(0.816, 3.134) | 1.782(1.052, 3.017) | 0.099 | 0.127 |
| Model 2 | 1 | 2.121(1.062, 4.233) | 2.042(1.196, 3.486) | 0.027 | 0.019 |
| Model 3 | 1 | 2.634(1.191, 5.822) | 3.101(1.709, 5.628) | 0.001 | 0.004 |
| Model 4 | 1 | 2.418(1.051, 5.564) | 2.790(1.489, 5.227) | 0.006 | 0.014 |
| Model 5 | 1 | 2.127(0.915, 4.943) | 2.531(1.351, 4.744) | 0.015 | 0.034 |

Model 1 did not adjust for confounding factors. Model 2 adjusted for age and gender. Model 3 adjusted for body mass index and waist circumference on the basis of Model 2. Model 4 adjusted for hypertension, pulse rate, alanine transaminas, aspartate transaminase, albumin, globulin, direct bilirubin, indirect bilirubin, blood urea nitrogen, serum uric acid, creatinine, triglycerides, high-density lipoprotein cholesterol, low-density lipoprotein cholesterol, fasting blood glucose, and homocysteine, on the basis of Model 3; Model 5 adjusted for weight maintenance in addition to the variables adjusted for in Model 4.


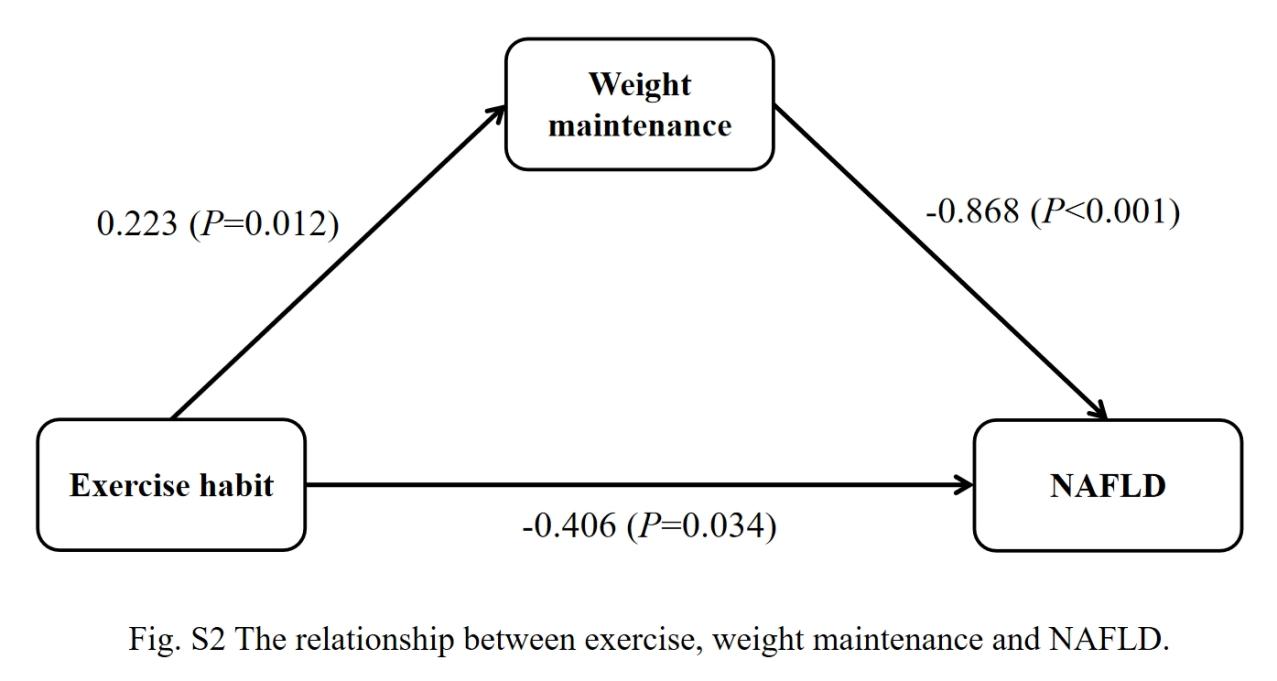


**Table S6 effect parameters calculated by product method**

| **Comparison** | **β_(X-M)_** | **β_(M-Y)_** | **β_(X-Y)_**  **Indirect effect** | **Direct effect** | **β_(X-Y)_**  **Total effect** | **Direct effect %** | **Indirect effect %** |
| --- | --- | --- | --- | --- | --- | --- | --- |
| non-exercise vs. moderate to high | -0.431 | -0.846 | 0.365 | 0.755 | 1.120 | 67.4 | 32.6 |
| low vs. moderate to high | -0.282 | -0.846 | 0.239 | 0.929 | 1.168 | 79.6 | 20.4 |
| no dummy variables | 0.223 | -0.868 | -0.194 | -0.406 | -0.600 | 67.7 | 32.3 |

**Table S7 effect parameters calculated by difference method**

| **Comparison** | **β_(X-M)_** | **β_(M-Y)_** | **β_(X-y)_**  **Indirect effect** | **Direct effect** | **β_(X-y)_**  **Total effect** | **Direct effect %** | **Indirect effect %** |
| --- | --- | --- | --- | --- | --- | --- | --- |
| non-exercise vs. moderate to high | -0.431 | -0.846 | 0.128 | 0.755 | 0.883 | 85.5 | 14.5 |
| low vs. moderate to high | -0.282 | -0.846 | 0.097 | 0.929 | 1.026 | 90.5 | 9.5 |
| no dummy variables | 0.223 | -0.868 | -0.058 | -0.406 | -0.464 | 87.5 | 12.5 |
